# EARLY DETECTION IN ALZHEIMER’S DISEASE AT ADVENTHEALTH: A DAVOS ALZHEIMER’S COLLABORATIVE FLAGSHIP SITE

**DOI:** 10.1093/geroni/igad104.2748

**Published:** 2023-12-21

**Authors:** Steven Smith, Richard Pratley, Valeria Baldivieso, Magda Baksh, Gayle Shepherd, Janice Lopez, Katherine Selzler, James Murray

**Affiliations:** AdventHealth, Orlando, Florida, United States; AdventHealth, Orlando, Florida, United States; Adventhealth, Orlando, Florida, United States; AdventHealth, Orlando, Florida, United States; AdventHealth, Altamonte Springs, Florida, United States; AdventHealth, Altamonte Springs, Florida, United States; Davos Alzheimer Collaborative, Wayne, Pennsylvania, United States; Davos Alzheimer’s Collaborative, Wayne, Pennsylvania, United States

## Abstract

**Background:**

The emergence of new therapeutics highlights the need for more efficient and equitable early identification of people at risk of developing Alzheimer’s disease. The Davos Alzheimer’s Collaborative flagship program was designed to test feasibility, acceptability, and implementation of a digital cognitive assessment (DCA) followed by a blood-based biomarker (BBM) as part of primary care workflows.

**Methods:**

Patients ≥65 years were contacted via their AdventHealth primary care provider or through social media to complete the Cogstate Brief Battery (CBB) DCA virtually. The CBB assesses four cognitive domains: processing speed, attention, visual learning, and working memory. Patients with an abnormal or borderline composite score were offered the PrecivityAD® blood test from C2N Diagnostics, an Alzheimer’s disease screening tool.

**Results:**

Approximately 2387 patients expressed interest to participate and 1077 e-consented: 96% social media, 4 % PCP. Of those, 742 patients completed the DCA. 28% were positive, 15% were borderline, and 56% were negative for cognitive issues. Additionally, 192 patients received the blood test results where, 17.7% had high likelihood, 67.7% low likelihood, and 14.6% could not distinguish presence or absence of amyloid plaques. Utilization of digital tools varied across race and ethnicity.

**Conclusion:**

Both PCP referrals and social media approaches demonstrated opportunities and challenges for early detection of Alzheimer’s. Implementation of both the CBB cognitive assessment and PrecivityAD® blood test was successful. Both DCAs and BBMs have value in early detection for cognitive impairment and a first step in identifying patients who could benefit from disease-modifying treatments.

